# Combined Probiotic *Lactiplantibacillus plantarum* ECGC 13110402 and Plant Sterol Supplement May Improve Lipids and Gut Microbiota in Coeliac Adults: A Randomised, Placebo-Controlled Pilot Human Intervention Study

**DOI:** 10.3390/molecules31101722

**Published:** 2026-05-19

**Authors:** Adele Costabile, Lorretta Olu Fagbemi, Carlo Soldaini, Monica Siniscalchi, Monica Ruotolo, Monica Barone, Marco Fabbrini, Patrizia Brigidi, Silvia Turroni, Sofia Kolida, Yvonne Jeanes, Carolina Ciacci

**Affiliations:** 1 School of Life and Health Sciences, University of Roehampton, London SW15 5PU, UK; 2 Department of Medicine, Surgery and Dentistry Scuola Medica Salernitana, University of Salerno, 84081 Fisciano, Italy; 3 UOC GSSS/PL Servizio di Dietetica e Nutrizione Clinica, Via Antonio Cardarelli, 80131 Napoli, Italy; 4 Human Microbiomics Unit, Department of Medical and Surgical Sciences, University of Bologna, 40126 Bologna, Italy; 5 IRCCS Azienda Ospedaliero-Universitaria di Bologna, 40138 Bologna, Italy; 6 Unit of Microbiome Science and Biotechnology, Department of Pharmacy and Biotechnology, University of Bologna, 40138 Bologna, Italy; 7 OptiBiotix Health PLC, York YO10 5DG, UK; 8 Education, Research & Professional Practice Team-British Dietetic Association, Birmingham B3 2TA, UK

**Keywords:** coeliac disease, probiotics, *Lactiplantibacillus plantarum* ECGC 13110402, plant sterols, gut microbiota, lipid profile, cardiometabolic health, dyslipidemia

## Abstract

Evidence suggests that a gluten-free diet may increase the risk of metabolic abnormalities associated with cardiovascular disease in adults with Coeliac Disease (CeD). This 9-week, double-blind, placebo-controlled, randomised pilot study investigated the effects of a combined supplement containing probiotic *Lactiplantibacillus plantarum* ECGC 13110402 and plant sterols and stanols, on cardiometabolic biomarkers and gut microbiota diversity and composition in adults with CeD and hypercholesterolaemia. Blood lipid profiles and vitamin D concentrations were analysed, and gut microbiota was profiled via 16S rRNA amplicon sequencing. In the active group, significant reductions in total cholesterol, LDL-cholesterol, non-HDL cholesterol, and apolipoprotein B were observed at multiple time points during the treatment phase, with changes generally greater in magnitude compared with the placebo group. Vitamin D levels also increased in the active group during supplementation. Microbiota analysis revealed potentially beneficial changes in participants receiving the active formulation, including higher alpha diversity and higher proportions of *Bifidobacterium* spp., *Christensenellaceae* R-7 group, and *Lachnospiraceae* ND3007 group. Overall, this feasibility study provides exploratory findings that a combined *Lactiplantibacillus plantarum* ECGC 13110402-phytosterol formulation may support lipid management and beneficially modulate gut microbiota in adults with CeD, particularly for those seeking non-pharmacological approaches to improving cardiometabolic health biomarkers.

## 1. Introduction

Coeliac Disease (CeD) is a chronic, immune-mediated disorder triggered by the ingestion of gluten, a protein complex found in wheat, barley, and rye. In genetically predisposed individuals carrying HLA-DQ2 and/or HLA-DQ8 alleles, gluten exposure triggers an abnormal immune response targeted at the mucosal lining of the small intestine, leading to villous atrophy and impaired nutrient absorption [1,2,3]. Lifelong adherence to a strict gluten-free diet (GFD) remains the only effective treatment and typically results in mucosal recovery and symptom resolution. However, concerns have been raised about the long-term nutritional adequacy of the GFD. Several studies report nutritional imbalances commonly associated with GFDs, including deficiencies in fibre, B vitamins, iron and calcium [4,5,6,7,8]. At diagnosis, CeD patients often show remarkably low cholesterol levels [9]. Observational studies additionally suggest that subsequent adherence to a GFD may paradoxically increase the risk of metabolic syndrome, including hypertension, obesity, insulin resistance, and dyslipidaemia—conditions that contribute to cardiovascular disease (CVD) risk [10,11,12,13]. While the direct relationship between CeD and CVD remains unclear, chronic inflammation, immune activation, oxidative stress, and micronutrient deficiencies in adults with CeD may contribute to atherogenesis and increased CVD risk [13,14].

The human gut microbiome, a complex and dynamic microbial ecosystem that inhabits our gastrointestinal tract, has undoubtedly emerged as a key factor in influencing our health, providing immune modulation, metabolic regulation, and nutrient synthesis, among other functions [15]. Dysbiosis, or an imbalance in its composition, is increasingly recognised as a contributing factor in CeD pathogenesis: microbiota shifts precede disease onset in genetically at-risk infants [16]. While genetic susceptibility (HLA-DQ2/DQ8) is necessary for disease onset, it is not sufficient, pointing to additional environmental and microbial triggers [2,17]. A recent study by Catassi et al. [18] emphasized the role of dysregulated immune responses originating from a disrupted microbiota in the initiation and progression of CeD. Seiler et al. [19] identified dysbiosis as a consistent feature of active CeD, and persistent microbial imbalances have also been observed in patients with refractory CeD—even among those strictly adhering to a GFD [20]. These findings underscore the potential role of the gut microbiota both in CeD pathogenesis and in influencing systemic outcomes, including cardiometabolic health.

Probiotics have emerged as a potential adjunct strategy to modulate the gut microbiota and support cardiometabolic health. However, a recent systematic review by Fagbemi et al. [21] found no studies evaluating probiotic-based cholesterol-lowering strategies specifically in CeD populations, a group with unique nutritional challenges and heightened cardiometabolic concerns. *Lactiplantibacillus plantarum* ECGC 13110402 is a well-characterized probiotic strain isolated from tomato juice. It was selected out of a collection of 353 lactobacilli for its superior bile salt hydrolase and cholesterol assimilation activity in vitro. It was further shown to significantly reduce serum LDL-cholesterol through improved bile acid metabolism and reduced cholesterol absorption in clinical trials in adults with hypercholesterolemia [22,23].

Several pharmacological therapies are available to support cholesterol management, including statins, fibrates, selective cholesterol-absorption inhibitors, and bile-acid sequestrants [24]. However, long-term adherence to lipid-lowering medication is suboptimal—around 50% in primary prevention and 66% in secondary prevention of cardiovascular disease [24]. Poor treatment compliance is often linked to adverse effects and patient perceptions about medication risks and necessity. This gap between clinical recommendations and real-world practice has increased interest in evidence-based non-pharmacological strategies that can complement conventional treatment.

Alongside probiotics, plant sterols and stanols (PSS) are well established for their LDL-cholesterol–lowering properties. PSS represent a promising adjunctive option. Structurally similar to cholesterol, they reduce intestinal cholesterol absorption through competitive inhibition, leading to clinically meaningful decreases in LDL-cholesterol. A substantial body of evidence indicates that daily consumption of 1.5–3 g lowers LDL-cholesterol by roughly 7–12% [25,26,27]. Recognising this effect, the European Food Safety Authority supports the inclusion of PSS-enriched foods as part of dietary strategies to improve lipid profiles [28]. Given their favourable safety profile and ease of incorporation into habitual diets, PSS may provide a practical non-pharmacological alternative or complement for individuals facing challenges with medication adherence.

Although the LDL-cholesterol–lowering efficacy of PSS is well established, current evidence does not conclusively demonstrate that these improvements translate into reduced long-term cardiovascular-event risk. Importantly, the absence of conclusive outcome data does not imply harmful effects but rather reflects the lack of sufficiently powered long-term trials specifically designed to evaluate cardiovascular endpoints. As noted in previous work, this creates an opportunity for supplements such as PSS to help address practical challenges in lipid management—particularly for individuals who are unwilling or unable to achieve optimal lipid control through medication alone—while acknowledging that they are best positioned as complementary, not substitutive, strategies within a broader cardiovascular-risk-reduction framework.

The selection of *L. plantarum* ECGC 13110402 and PSS was based on their distinct but potentially complementary cholesterol-lowering mechanisms. *L. plantarum* ECGC 13110402 has previously demonstrated lipid-lowering effects, largely attributed to bile salt hydrolase activity and consequent modulation of bile acid metabolism. In contrast, PSS reduce cholesterol absorption within the intestinal lumen by competing with cholesterol for incorporation into mixed micelles. Their combination was therefore intended to target two key aspects of cholesterol homeostasis—intestinal absorption and enterohepatic bile acid cycling—within a single intervention strategy.

In this pilot study, we evaluated a combined formulation containing *L. plantarum* ECGC 13110402 and PSS in hypercholesterolaemic adults with CeD. The study aimed to: (i) examine the feasibility, acceptability, and safety of this dual-ingredient supplement in a CeD population, and (ii) explore its effects on blood lipid profiles and gut microbiota diversity and composition. This study represents an important first step in addressing the unmet need for adjunctive strategies to support cardiometabolic and intestinal health in adults with CeD beyond dietary management alone.

## 2. Materials and Methods

### 2.1. Study Design, Setting and Ethics

This pilot, randomized, double-blind, placebo-controlled study was conducted at the UOC of Gastroenterology of the AOU Ruggi d’Aragona of Salerno (Italy). The trial was registered at Clinical Trials.gov (ID: NCT06178107) and carried out in accordance with the Declaration of Helsinki and Good Clinical Practice (GCP) guidelines. Ethical approval for the study protocol was obtained from the Campania Sud Ethics Committee (CECS; opinion no. 133_r.psc, 7 September 2022), the NHS Ethics Committee in Italy (Ref. 115 Del 18 October 2022), the University of Roehampton Research Ethics Committee (ref.: LSC18/241), and the NHS Research Ethics Committee (IRAS project ID: 259363). The study complied with the requirements of good clinical practice (DMS of 15 July 1997), the Ministerial Decree of 17 December 2004 concerning non-profit clinical studies, and the Declaration of Helsinki. All participants were screened against the inclusion and exclusion criteria (Table 1) prior to providing written informed consent.

### 2.2. Study Participants

**Figure 1:**
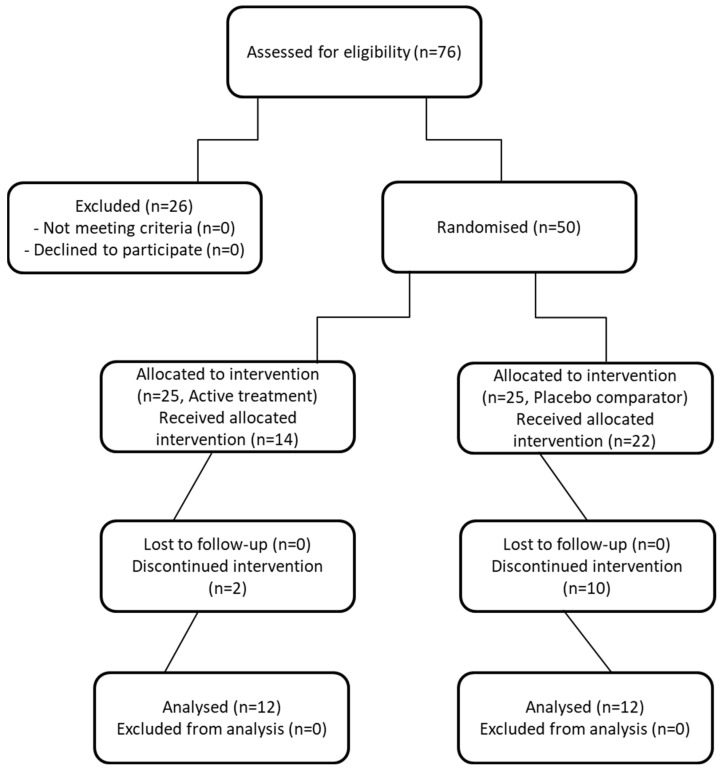
Flow of study participants through the intervention.

Study participants were recruited through telephone contact, social media advertisements, and direct engagement during clinic visits at the AOU Ruggi d’Aragona of Salerno (Italy). Recruitment was carried out between January and May 2023. Volunteers were asked to read the Participant Information Sheet (PIS) and subsequently provide written informed consent. Only after consent was obtained were volunteers asked to complete the general screening questionnaires used to assess their eligibility for the study and invited to a screening visit during which anthropometric measurements and fasting blood cholesterol levels were assessed. Participants were stratified by gender and randomly assigned to the active or placebo group using a 1:1 ratio, reflecting the greater prevalence of females with CeD (Figure 1). Study personnel and participants remained blinded to group allocation until completion of primary data analysis. Randomization of treatment sequence and allocation was achieved by an independent researcher using a random number generator (GraphPad QuickCalcs, San Diego, CA, USA). Each participant was given a code ID to help ensure all allocation information remained concealed throughout the study.

### 2.3. Intervention

The study intervention comprised a 9-week treatment phase followed by a 3-week washout period. Study visits were conducted at baseline, week 5 (mid-intervention), week 9 (end of treatment) and week 12 (WO, washout). During the intervention phase, participants were instructed to take one capsule twice daily with a main meal and to maintain their usual dietary intake and physical activity levels. The active supplement was formulated as a bilayer tablet containing 50 mg of *Lactiplantibacillus plantarum* ECGC 13110402 (2 × 10^9^ colony-forming units (CFU)/tablet) in one layer, and 400 mg of PSS (i.e., beta-sitosterol, campesterol, stigmasterol, brassicasterol, and sitostanol) in the other layer.

*Lactiplantibacillus plantarum* ECGC 13110402 is a previously characterised probiotic strain selected for its documented bile salt hydrolase activity and cholesterol-lowering potential. The plant sterol and stanol fraction used in the active formulation consisted of a defined mixture of phytosterols/phytostanols, including beta-sitosterol, campesterol, stigmaserol, brassicasterol, and sitostanol, rather than an undefined crude extract. The probiotic was incorporated into the bilayer tablet in a stabilised dried form, while the second layer contained the plant sterol/stanol component. The placebo contained identical excipients but omitted *L. plantarum* ECGC 13110402 and PSS. Both tablets were visually indistinguishable, packaged identically, and supplied free of charge.

Study participants were asked to take a tablet with a main meal twice daily and were instructed to avoid other probiotic products, phytosterol-containing foods (e.g., fortified spreads), and medications that could influence gastrointestinal motility or lipid metabolism. Compliance was monitored through food diaries and tablet counts.

### 2.4. Anthropometric Measurements

Body measurements taken during all visits included weight, height, and waist and hip circumferences, based on Lohman et al. [29] standard techniques. Participants stood upright and shoe-free while measuring their height using a stadiometer (Seca Ltd., London, UK) to the closest centimetre. Body weight was recorded to the nearest 0.1 kg without shoes. The conventional formula for calculating body mass index (BMI) was weight (kg)/height (m)^2^. A Tanita BC-418 MA (Tanita UK Ltd., Middlesex, UK) body composition analyser was used to calculate the percentage of body fat.

### 2.5. Dietary Intake

Nutritional data were collected at baseline and at week 12 for both groups using a 7-day food diary (i.e., seven days after the intervention began and seven days before it ended). From the baseline visit through to the study completion, all participants were advised to refrain from consuming any prebiotics and fermented foods as part of their diet. Compliance monitoring was conducted through structured weekly calls with the dietitians, during which dietary intake and adherence to the supplementation protocol were reviewed and recorded. During these sessions, participants were asked to report their intake, and adherence was cross-checked against supplementation logs and self-reported compliance records to ensure consistency and completeness of reporting. Nutrient intake was calculated by a dietitian using food composition tables specific to typical Italian foods, including their nutrient content and portion sizes. No nutritional analysis software was used. The reference tables were obtained from the Italian National Food Composition Database (available at https://www.alimentinutrizione.it/sezioni/tabelle-nutrizionali accessed on 14 May 2026). Nutrients assessed included protein (g), carbohydrates (g), lipids (g), sugars (g), fibre (g), saturated fatty acids (g), cholesterol (mg), and total energy intake (kcal).

### 2.6. Blood Sample Collection and Biochemistry Analyses

Fasted venous blood samples were collected from the antecubital vein using a sterile 21-gauge venepuncture set, in accordance with the World Health Organization (WHO) [30] standard protocol. Samples were drawn into 12 mL collection tubes containing lithium heparin, ethylenediaminetetraacetic acid (EDTA), or sodium fluoride/potassium oxalate (BD Vacutainer^®^, Cowley, Oxfordshire, UK), depending on the analyte to be measured. Plasma and serum were analysed for fasting total cholesterol (TC) (desirable range: <5.2 mmol/L), low-density lipoprotein cholesterol (LDL-C) (optimal: <3.0 mmol/L), high-density lipoprotein cholesterol (HDL-C) (≥1.0 mmol/L in men; ≥1.3 mmol/L in women), triglycerides (TG) (<1.7 mmol/L), aspartate aminotransferase (AST) (10–40 U/L), alanine aminotransferase (ALT) (7–56 U/L), apolipoprotein B (ApoB) (0.6–1.2 g/L), vitamin D (25(OH)D) (sufficient: ≥50 nmol/L), total protein (60–80 g/L), total bilirubin (3–21 µmol/L), and albumin (35–50 g/L). Prior to centrifugation, samples were stored on ice. Blood was centrifuged at 1700× *g* for 10 min at 4 °C. The separated plasma or serum was aliquoted into 1.5 mL microcentrifuge tubes and stored at −80 °C until analysis. Biochemical parameters were measured using the Daytona automated clinical chemistry analyser (Randox Laboratories Ltd., Antrim, UK).

### 2.7. Stool Sample Collection and Microbiota Profiling

Participants were provided with home stool sample collection kits (OM-200 microbiome stool kits; DNA Genotek Inc., Ottawa, ON, Canada) and were given instructions on how to collect and safely store stool samples before their visits. Stool samples were collected at each study visit (baseline and weeks 5, 9 and 12). All samples were stored at −80 °C until processing.

Microbial DNA was extracted from 250 mg of faecal sample using the QIAamp DNA Stool kit (QIAGEN, Hilden, Germany), in accordance with the manufacturer’s instructions. A Nanodrop-One spectrophotometer (Thermo Fisher Scientific, Waltham, MA, USA) was used to measure DNA concentration. Amplicon sequencing of 16S rRNA was performed as reported by Costabile et al. [31]. Briefly, the V3-V4 hypervariable regions of the 16S rRNA gene were amplified using the S-D-Bact-0341-b-S-17 and S-D-Bact-0785-a-A-21 primers [32], incorporating Illumina overhang adapter sequences. PCR was conducted using a SimpliAmp Thermal Cycler (Thermo Fisher Scientific). PCR products were purified using magnetic bead-based technology (Agencourt AMPure XP; Beckman Coulter, Brea, CA, USA) and then used to construct indexed libraries via limited-cycle PCR using Nextera technology, followed by further purification. Equimolar amounts of the final libraries were pooled (4 nM), denatured with 0.2 N NaOH, and diluted to 6 pM prior to sequencing on an Illumina MiSeq platform utilizing a 2 × 250 bp paired-end protocol as per manufacturer’s instructions (Illumina, San Diego, CA, USA). The sequencing reads were deposited in the National Center for Biotechnology Information Sequence Read Archive (Bioproject ID: PRJNAXXX).

A customized bioinformatic pipeline was employed to process the raw sequence data, as described in Fabbrini et al. [33] and available at https://github.com/FabbriniMarco/16S-KrakenBracken-Pipeline (accessed on 14 May 2026). This included quality control of the raw reads using the fastp tool [34], followed by the exclusion of host-derived sequences through alignment to the human genome reference (hg38). Non-host reads were subsequently classified against the SILVA 138.1 SSURef NR99 database [35] using Kraken2 [36]. Taxonomic assignment was refined using Bracken [37], following the developer’s instructions. Measures of alpha diversity, both phylogenetic and non-phylogenetic, were computed using the otuSummary R package [38]. Beta diversity was assessed using UniFrac distance measurements implemented via the UniFrac function in the phyloseq package [39].

### 2.8. Statistical Analysis

As this investigation was designed as a pilot feasibility study, the primary objectives were to evaluate the recruitment and retention processes, assess compliance with the intervention, determine the acceptability of study procedures, and generate preliminary estimates of variability in key clinical and microbiota outcomes. Consistent with feasibility methodology, the study was not powered to detect definitive between-group differences, meaning all statistical analyses were exploratory. GraphPad Prism version 9.0 (GraphPad Software, Inc., San Diego, CA, USA) was used for the analysis of clinical, dietary intake, and biochemical data, as well as for graphical presentation. Additional statistical analyses were performed using IBM SPSS Statistics (version 30; SPSS Inc., Chicago, IL, USA). Continuous variables were summarised as mean ± standard deviation. Changes in biochemical markers (vitamin D, total protein, AST, ALT, insulin, albumin, and bilirubin) over time were analysed using a two-way repeated-measures analysis of variance (ANOVA), with time (baseline, week 5, week 9, and week 12) as the within-subjects factor and group (treatment vs. placebo) as the between-subjects factor. Mauchly’s test was used to assess the assumption of sphericity, and where violations occurred the Greenhouse–Geisser correction was applied. The model examined the main effects of time and group as well as the time × group interaction. Effect sizes were reported as partial eta squared (η^2^p).

Changes in total cholesterol were further expressed as Δ (delta) total cholesterol, calculated as post-intervention minus baseline values. Moreover, the associations between changes in total cholesterol and other continuous variables were explored using Pearson correlation analysis. These analyses were interpreted with caution due to the small sample size typical of feasibility studies. Microbiota data were analysed using RStudio 2025.05.0+496 and R 4.5.0. Data visualization, including boxplots and Principal Coordinate Analysis (PCoA), was performed using the microbAIDeR package version 0.3.2 [40]. Beta diversity differences were assessed using permutational multivariate analysis of variance (PERMANOVA) via the adonis function in the vegan package. Graphical outputs were produced using the ggplot2 package [41]. Differences in alpha diversity and the relative abundance of taxa were evaluated using a Kruskal–Wallis test, followed by post hoc Wilcoxon tests. Statistical significance was defined as *p* ≤ 0.05 (two-sided *p* value), with *p* ≤ 0.1 being considered indicative of a trend.

## 3. Results

### 3.1. Subjects

Because this study was designed as a pilot trial, participant flow also reflected practical constraints such as scheduling, availability for follow-up visits, and willingness to commit to repeated sampling. Of the 50 participants initially enrolled, 26 participants were excluded from the final analysis. One participant withdrew due to low probiotic-intake compliance. Two participants failed to attend any of the follow-up visits. Six participants were discontinued due to personal matters. Five were lost to follow-up despite repeated contact attempts. Four were excluded because of protocol violations, and eight had incomplete outcome data. As a result, 24 participants were able to complete all visits and provided the full set of biological samples required for analysis (*n* = 12 per group). These numbers are consistent with feasibility research, in which the primary aim was to evaluate recruitment, retention, and protocol adherence rather than to achieve a predetermined sample size for statistical power. Thus, the participant flow reflected practical constraints such as scheduling, availability for follow-up visits, and willingness to commit to repeated sampling. Baseline characteristics of the 24 participants who completed all study procedures—including age, gender, height, and weight—are presented in Table 2. No differences were observed between the active and placebo groups. Because this study was designed as a pilot feasibility trial, participant flow also reflected practical constraints such as scheduling, availability for follow-up visits, and willingness to commit to repeated sampling.

### 3.2. Outcomes

#### 3.2.1. Blood Lipids and Inflammatory Biomarkers

**Figure 2:**
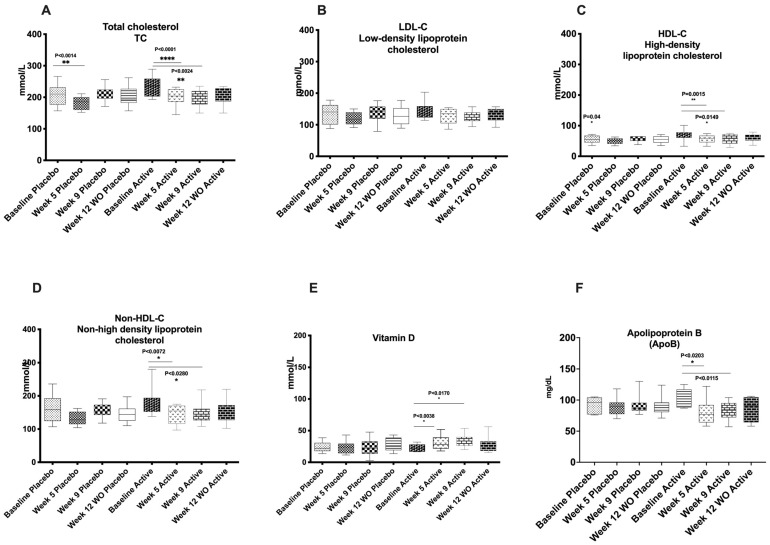
Biochemical parameters of participants in the active and placebo groups at baseline, week 5 (mid-intervention), week 9 (study completion) and week 12 (WO, wash-out). (**A**) Total cholesterol (TC); (**B**) Low-density lipoprotein cholesterol (LDL-C); (**C**) High-density lipoprotein cholesterol (HDL-C); (**D**) Non-high-density lipoprotein cholesterol (non-HDL-C); (**E**) Vitamin D (VitD); (**F**) Apolipoprotein B (ApoB). * *p* value < 0.05 (two-sided *p* value); ** *p* value < 0.01 (two-sided *p* value); **** *p* value < 0.001.

Figure 2 illustrates changes in biochemical parameters throughout the intervention. Total cholesterol levels decreased significantly in the placebo group at week 5 (mid-intervention) (from 207.5 ± 32.04 to 182.4 ± 20.82, 12.1% reduction from the baseline, *p* = 0.0104), but this reduction was not maintained at week 9 (from 207.5 ± 32.04 to 211.0 ± 23.13, 1.7% increase from the baseline to the end of intervention) or week 12 (from 207.5 ± 32.04 to 201.0 ± 31.62, 3.1% decrease at the end of the wash-out period). In contrast, the active group showed significant reductions in total cholesterol at all time points compared with baseline (from 236.1 ± 31.56 to 207 ± 27.20, 12.3% reduction from the baseline to week 5, *p* < 0.0001; from 236.1 ± 31.56 to 198.6 ± 27.09, 16% reduction from the baseline to week 9, *p* = 0.0024; from 236.1 ± 31.56 to 204.5 ± 25.76, 13.4% reduction from the baseline to the wash-out, *p* = 0.0003, respectively). The change in total cholesterol (ΔTC) between baseline (T0) and week 12 (T12) differed significantly between groups, with a greater reduction observed in the active group (−31.58 mg/dL) compared with the placebo group (−5.92 mg/dL). Pearson correlation analysis demonstrated a significant inverse association (r = −0.571, *p* = 0.004).

LDL-cholesterol concentrations did not change significantly in the placebo group at week 5 (from 133.9 ± 32.71 to 119.3 ± 21.22, 11% reduction from the baseline, *p* = 0.13) and remained unchanged at week 9 (from 133.9 ± 32.71 to 137.0 ± 27.85, 2.3% increase from the baseline, *p* = 0.86). In the active group, LDL-cholesterol showed greater reductions, reaching significance at week 5 (from 143.3 ± 26.08 to 128.3 ± 23.18, 10.5% reduction from the baseline, *p* = 0.05), although the change at week 9 was not statistically significant (from 143.3 ± 26.08 to 125.3 ± 19.86, 13% reduction from the baseline, *p* = 0.16).

Significant reductions were observed in HDL-cholesterol concentrations with both active and placebo treatments. Significant reductions were observed in the placebo group at week 5 (from 55.70 ± 12.59 to 49.50 ± 9.62, 11% reduction from the baseline, *p* = 0.04). In the active group, significant decreases were observed at week 5 (from 69.00 ± 16.48 to 55.33 ± 13.29, 20% reduction from the baseline to week 5, *p* = 0.0015) and week 9 (from 69.00 ± 16.48 to 57.82 ± 16.01, 16.2% reduction from the baseline to week 9, *p* = 0.015).

Both groups showed reductions in non-HDL cholesterol; however, significant decreases were observed only in the active group at week 5 (from 179.6 ± 40.72 to 145.3 ± 27.92, 19.1% reduction from baseline to week 5, *p* = 0.007) and week 9 (from 179.6 ± 40.72 to 145.6 ± 2984, 19% reduction from baseline to week 9, *p* = 0.028). Apolipoprotein B concentrations decreased significantly only in the active group at week 5 (from 103.06 ± 15.37 to 81.55 ± 21.22, 21% reduction from baseline to week 5, *p* = 0.019) and week 9 (from 103.06 ± 15.37 to 83.50 ± 14.23, 19% reduction from baseline to week 9, *p* = 0.023).

Triglyceride concentrations decreased in the active group at week 5 (from 90.83 ± 49.07 to 85.75 ± 41.24, 5.6% reduction from the baseline to week 5, *p* = 0.2), at week 9 (from 90.83 ± 49.07 to 81.55 ± 35.08, 10.2% reduction from the baseline to week 9, *p* = 0.6) and remained stable until week 12 (from 90.83 ± 49.07 to 81.75 ± 27.49, 10% reduction from baseline to the wash-out period, *p* = 0.87, although these changes were not statistically significant.

Finally, significant increases in serum total vitamin D concentrations were observed only in the active group at week 5 (from 22.74 ± 5.94 to 31.37 ± 11.08, 38% increase from the baseline to week 5, *p* = 0.019) and week 9 (from 22.74 ± 5.94 to 34.26 ± 9.99, 51% increase from the baseline to week 9, *p* = 0.017), whereas no significant changes were detected in the placebo group (from 24.21 ± 8.157 to 23.57 ± 9.98, 3% reduction from the baseline to week 5, *p* = 0.7 and from 24.21 ± 8.157 to 23.23 ± 13.08, 4% reduction from the baseline to week 9, *p* = 0.7). Moreover, there was a significant main effect of time for vitamin D levels (F(2.81, 19.66) = 3.69, *p* = 0.032, η^2^p = 0.345), indicating that vitamin D concentrations changed significantly across the study time points. However, the time × group interaction was not statistically significant (F(2.81, 19.66) = 2.63, *p* = 0.081), suggesting that the pattern of change over time did not differ significantly between the treatment and placebo groups. No significant main effect of group was observed (F(1, 7) = 0.04, *p* = 0.851).

#### 3.2.2. Biomarkers of Liver Function

Changes in biomarkers of liver function are reported in Table 3 and Table 4. A repeated-measures ANOVA was conducted to evaluate the effects of time and treatment group on biochemical markers over the 12-week study period. For the remaining biochemical markers, no significant main effects of time or group and no significant time × group interactions were observed. Specifically, total protein (time: *p* = 0.213; interaction: *p* = 0.834), AST (time: *p* = 0.138; interaction: *p* = 0.401), ALT (time: *p* = 0.386; interaction: *p* = 0.333), insulin (time: *p* = 0.770; interaction: *p* = 0.214), albumin (time: *p* = 0.321; interaction: *p* = 0.778), and bilirubin (time: *p* = 0.165; interaction: *p* = 0.160) showed no statistically significant differences over time or between treatment groups.

### 3.3. Gut Microbiota Profiling

**Figure 3:**
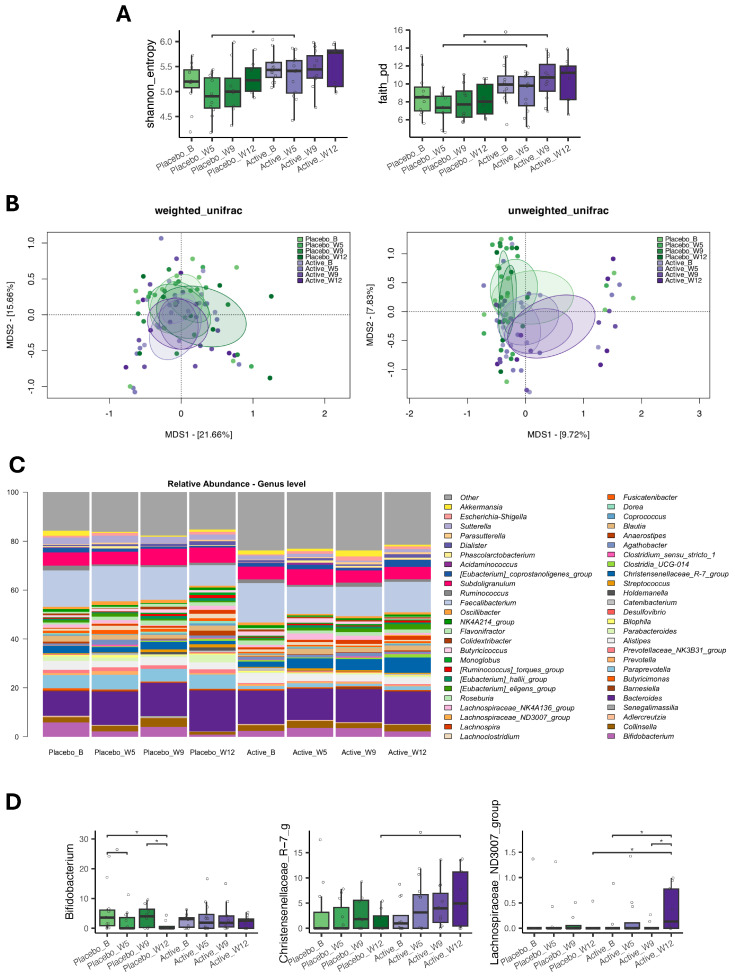
Gut microbiota diversity and composition in participants from the active and placebo groups at baseline, week 5 (mid-intervention), week 9 (study completion) and week 12 (WO, wash-out). (**A**) Boxplots showing the distribution of alpha diversity, computed with the Shannon index (**left**) and Faith’s phylogenetic diversity (**right**). Wilcoxon test, * *p* value ≤ 0.05; *p* value ≤ 0.1. (**B**) Principal Coordinates Analysis (PCoA) based on Weighted (**left**) and Unweighted (**right**) UniFrac distances between microbiota profiles. (**C**) Bar plots showing genus-level relative abundance profiles across samples. (**D**) Boxplots showing the relative abundance distribution of genera differentially represented between the study groups. Wilcoxon test, * *p* value ≤ 0.05; *p* value ≤ 0.1.

Alpha diversity was or tended to be higher at weeks 5 (*p* = 0.05, Wilcoxon test) and 9 (*p* value = 0.07) in the active group than in the placebo group (Figure 3A). Principal Coordinates Analysis (PCoA) of beta diversity revealed significant segregation between samples (*p* ≤ 0.05, PERMANOVA) (Figure 3B). In particular, the samples from the active and placebo groups were significantly separated at weeks 9 and 12 in the unweighted UniFrac-based PCoA plot.

Overall, the compositional profiles were not affected, with genera of the families *Ruminococcaceae*, *Lachnospiraceae* and *Bacteroidaceae* dominating the ecosystem at all time points (Figure 3C). However, some interesting trends emerged (Figure 3D). The relative abundance of *Bifidobacterium* decreased over time in the placebo group (*p* ≤ 0.1, Wilcoxon test) but remained stable in the active group. The proportions of the *Christensenellaceae* R-7 group tended to be higher in the active group than in the placebo group at week 12 (*p* = 0.06). Finally, the *Lachnospiraceae* ND3007 group increased significantly in the active group at week 12 compared to baseline, with levels significantly higher than in the placebo group (*p* ≤ 0.05).

### 3.4. Dietary Intake

No significant differences were observed in total daily energy intake or nutrient composition—including protein, carbohydrate, fat, sugar, fibre, saturated fatty acids, and cholesterol—between participants in the placebo and active groups (*p* > 0.05) (Table 5). Dietary fibre intake in both groups was below the recommended intake of 30 g per day for adults [42,43]. However, the active group demonstrated a slightly higher mean fibre intake (22 ± 10.8 g) compared with the placebo group (18.5 ± 2.86 g). These findings are consistent with population-level dietary surveys indicating that average fibre consumption in the UK and many Western populations remains below recommended levels [44,45]. Insufficient fibre intake has been widely reported in dietary studies and is associated with increased risk of several chronic diseases, including cardiovascular disease and type 2 diabetes [46,47].

## 4. Discussion

This pilot, randomised, placebo-controlled study evaluated the effects of a combined probiotic–phytosterol supplement containing *Lactiplantibacillus plantarum* ECGC 13110402 and PSS intervention on lipid profile, vitamin D status, and gut microbiota composition in adults with CeD and hypercholesterolaemia. Overall, the intervention was safe, well tolerated, and associated with improvements in atherogenic lipid markers alongside shifts in gut microbiota composition.

The cholesterol-lowering effect of *Lactiplantibacillus plantarum* ECGC 13110402 has previously been demonstrated and is thought to be mediated primarily through its bile salt hydrolase activity [22,23]. However, to date, this mechanism and its associated clinical effects have not been specifically investigated in adults with CeD. PSS are also well established for their cholesterol-lowering effects through competitive inhibition of intestinal cholesterol absorption. However, the efficacy of a joint treatment has not been previously investigated. The lipid-modulating effects observed in the present study are therefore likely to reflect the complementary mechanisms of both components and are consistent with previous studies reporting reductions in total cholesterol, LDL-cholesterol, non-HDL cholesterol, and apolipoprotein B following supplementation with *L. plantarum* strains or PSS individually.

As a feasibility study, this trial also provided important information regarding recruitment, retention, and intervention acceptability, while generating preliminary biomarker data to support further investigation. Secondary analyses examined potential modulation of the gut microbiota in the context of a GFD and probiotic supplementation, as well as associations with dietary intake, lifestyle factors, and metabolic markers, including liver function parameters and vitamin D status.

LDL-cholesterol decreased consistently in the active group compared with the placebo group, with sustained reductions during treatment and partial rebound during washout, suggesting a treatment-dependent effect. Similar trends were observed for non-HDL-cholesterol and apolipoprotein B, supporting a reduction in atherogenic lipoprotein burden.

Serum total vitamin D concentrations increased progressively during the treatment phase in the active group, aligning with existing evidence that alterations in both 25(OH) and 1,25(OH) vitamin D are common in adult coeliac disease and may improve with treatment [48,49]. As this study was conducted in Italy, beginning in winter and concluding in late July, seasonal variation in sunlight exposure is likely to have contributed to the overall rise in vitamin D levels. Increased cutaneous synthesis due to greater ultraviolet B (UVB) exposure from spring to summer is well documented, particularly in Mediterranean regions [50,51]. However, if seasonal exposure were the sole determinant, a comparable increase would be expected in both the active and placebo groups. The observation that statistically significant increases were detected only in the active group suggests that, in addition to seasonal variability, a potential treatment-related effect cannot be excluded.

Indirect mechanisms related to the intervention may have contributed to this observation. Vitamin D is a fat-soluble micronutrient whose intestinal absorption depends on efficient bile acid–mediated micelle formation. *L. plantarum* ECGC 13110402 may influence bile acid metabolism through its bile salt hydrolase activity, which could potentially enhance the absorption of fat-soluble vitamins [52,53]. This mechanism may be particularly relevant in individuals with coeliac disease (CeD), in whom subtle nutrient malabsorption can persist despite long-term adherence to a gluten-free diet [54,55]. Previous studies [56] further suggest that *L. plantarum* strains can increase vitamin D receptor (VDR) expression, resulting in an increase in vitamin D absorption. While the present study was not designed to evaluate vitamin D absorption specifically, the differential change observed between groups raises the possibility that the intervention may have facilitated improved vitamin D uptake in conjunction with seasonal increases in UVB exposure. Although serum vitamin D concentrations increased significantly within the active group, this finding should be interpreted cautiously because the time × group interaction did not reach statistical significance. Seasonal variation in sunlight exposure may have contributed to the overall increase. Nevertheless, the absence of a comparable significant change in the placebo group suggests that a possible intervention-related contribution cannot be excluded and warrants further investigation.

Potentially beneficial changes related to the treatment were also observed in gut microbiota diversity and composition. Specifically, the active group showed higher alpha diversity—a hallmark of intestinal and systemic health—and increased proportions of several key genera. These included *Bifidobacterium* [57,58,59], the *Christensenellaceae* R-7 group, and the *Lachnospiraceae* ND3007 group. The microbial changes observed in the active group may have potential clinical relevance, although they remain exploratory. Preservation of *Bifidobacterium* and increases in *Christensenellaceae* R-7 and *Lachnospiraceae* ND3007 groups may indicate a shift toward a more saccharolytic microbiota. These taxa are associated with saccharolytic fermentation and short-chain fatty acid production, metabolites that play a multifactorial role in host physiology [60], support gut barrier integrity, and promote metabolic and cardiovascular health, including cholesterol reduction and healthy aging [61]. However, the present study relied on 16S rRNA profiling and did not include metagenomics, metabolomics, or bile acid analyses. Therefore, specific metabolic pathways linking these taxa to cholesterol reduction or PSS metabolism cannot be confirmed from the present data. Although the separation in beta diversity and the increase in *Lachnospiraceae* ND3007 and *Christensenellaceae* R-7 groups may be consistent with intervention-related microbiota modulation, the present study cannot determine whether these changes were driven primarily by the probiotic, PSS, or their combination. These taxa may be involved in saccharolytic fermentation, short-chain fatty acid production, bile acid metabolism, or sterol-related microbial processes, but these pathways were not directly measured. Future studies should therefore include functional metagenomics, faecal short-chain fatty acids, bile acid profiling, and sterol metabolite analyses. Studies consistently show that higher levels of these bacteria are also associated with positive health outcomes in conditions such as obesity and inflammatory bowel disease. Interestingly, their presence appears to be influenced by genetics, although the exact genes involved are still unclear [61,62,63,64]. Importantly, in the context of CeD, these changes may help mitigate intestinal inflammation, enhance mucosal healing, and improve overall gastrointestinal function, potentially complementing the benefits of a GFD. Such variations are likely to be closely related to PSS, for which a beneficial modulatory effect on the gut microbiota has already been demonstrated [65,66,67,68]. In particular, PSS could stimulate the growth of bacteria endowed with sterol-metabolizing capacity, including the above taxa. Previous studies [69,70] have also found an association between the aforementioned taxa and vitamin D, whose increase in the active group preceded the microbial shifts.

The intervention was safe and well tolerated, with no adverse gastrointestinal effects. Despite the study limitations related to the small sample size, its short duration, and the lack of statistical power, these findings provide encouraging evidence supporting further investigation of *L. plantarum* ECGC 13110402 as an adjunct strategy for lipid and cardiovascular risk management in adults with CeD. The outcomes of the study suggest a benefit in combining *L. plantarum* ECGC 13110402 and 800 mg/day PSS, two active ingredients with complementary mechanisms of action for cholesterol reduction, for the population under investigation. Albeit a pilot study, the improvements observed in the lipid profiles are significantly higher compared to those previously attributed to the daily intake of 1.5–3.0 g PSS. Consumption of 2–3 g PSS daily has been associated with reductions in ApoB of 4–10% [71,72]. ApoB is a key atherogenic biomarker, and a well-recognised risk factor for vascular diseases [73]. Our findings report reductions in ApoB of 19% with the active treatment, aligned with previous studies on the intake of *L. plantarum* ECGC 13110402 in hypercholesterolaemic adults [23]. Additionally, compared to baseline levels, total cholesterol was statistically significantly reduced by 12.3% in the active group at week 5, 16% at week 9 and 13.4% at the end of wash-out. LDL cholesterol was significantly reduced by 11%. Previous studies suggest decreases in LDL-cholesterol of 5–12% with the consumption of higher PSS doses of 1.5–3 g/d [28]. The transient reduction in total cholesterol observed in the placebo group at week 5 was not maintained at week 9 or after washout.

This pattern suggests that the early decrease may have reflected short-term behavioural changes or regression to the mean rather than a sustained biological effect of placebo. Although participants were instructed to maintain their usual diet and physical activity, regular contact with the study team may have temporarily influenced dietary choices, particularly during the early phase of the intervention.

The non-HDL cholesterol and ApoB reductions observed in the study are considerably higher than those previously associated with 1.5–3 g/d PSS intake. The active treatment was formulated to deliver a total of 2 × 10^9^ CFU/day/twice and 800 mg/d PSS in two doses taken with the main meals of the day to align with the mechanism of action of PSS for cholesterol reduction and the timing of maximum bile salt circulation, ensuring optimum efficacy for both actives in the treatment. The previously unreported large reductions in total cholesterol, non-HDL cholesterol and ApoB observed in the active group may therefore reflect the combined targeting of intestinal cholesterol absorption and bile acid metabolism. Nevertheless, because this was a small exploratory pilot study, these findings should be interpreted cautiously and confirmed in adequately powered trials.

Although a substantial body of research has explored alterations in the gut microbiota in CeD, there remains uncertainty about whether these microbial changes are a consequence of the disease itself or whether they actively contribute to its onset and progression [74,75]. Current evidence does not yet allow a definitive distinction between cause and effect, highlighting an important gap in understanding the microbiota–CeD relationship. Nevertheless, it is increasingly recognised that gut dysbiosis may play a functional role in perpetuating low-grade inflammation, impairing intestinal barrier integrity, and influencing systemic metabolic pathways. In adults with CeD, these processes may become particularly relevant in the context of a long-term gluten-free diet (GFD). While the GFD is essential for mucosal healing, it is often characterised by reduced intake of dietary fibre and micronutrients, alongside increased consumption of ultra-processed gluten-free products that are frequently higher in saturated fats, sugars, and refined starches. Evidence from studies on gut microbiome–metabolism interactions indicates that restoring microbial homeostasis can positively influence lipid handling, including reductions in low-density lipoprotein (LDL) cholesterol and improvements in overall metabolic status. This is particularly significant given the established association between CeD, metabolic dysfunction-associated steatotic liver disease (MASLD), and dyslipidaemia, which collectively amplify CVD risk. Clinically, these findings support a more integrated approach to cardiovascular risk management in CeD. Beyond strict adherence to a gluten-free diet, which remains foundational, there is growing justification for longitudinal monitoring of metabolic, hepatic, and lipid parameters. In addition, adjunctive strategies targeting gut microbiota modulation—such as personalised dietary fibre optimisation, prebiotic and probiotic interventions, and broader biotic-based therapies—may offer potential benefits in improving metabolic health. Although evidence is still emerging, these approaches align with current understanding of the gut–metabolism–immune axis and may represent a promising direction for reducing long-term cardiovascular risk in adults with CeD [76]. From a clinical perspective, the combined improvements in lipid profiles and gut microbiota may have practical implications for cardiovascular risk management in adults with CeD [74,76]. Reductions in atherogenic lipid markers such as LDL-cholesterol, non-HDL cholesterol, and apolipoprotein B are directly relevant to risk stratification and established therapeutic targets in clinical practice. Concurrent microbiota modulation may further support metabolic regulation through pathways involving short-chain fatty acid production, bile acid metabolism, and systemic inflammation. Although preliminary, these findings suggest that microbiota-targeted interventions may complement traditional dietary and pharmacological approaches, offering a potential adjunctive strategy for improving cardiometabolic health in this population.

Several limitations should be acknowledged. This pilot feasibility study was not powered to establish definitive efficacy; therefore, all statistical findings should be considered exploratory. The relatively short intervention duration also limits conclusions regarding long-term lipid control, microbiota stability, and cardiovascular risk reduction. Although the gender distribution differed between groups, this likely reflects the higher prevalence of CeD in women. The substantial dropout rate may have introduced selection bias, as participants completing the study may have been more able or motivated to adhere to repeated visits and sampling procedures; however, attrition itself also represents an important feasibility outcome. Future adequately powered randomised controlled trials should include larger and more balanced cohorts, longer follow-up, detailed dietary assessment, and functional analyses, including bile acid profiling, sterol absorption markers, metagenomics, and metabolomics.

In conclusion, this pilot feasibility study suggests that a combined *L. plantarum* ECGC 13110402 and PSS formulation is safe, well tolerated, and acceptable in adults with CeD and hypercholesterolaemia. Despite the exploratory design, the intervention was associated with consistent improvements in atherogenic lipid markers, including LDL-cholesterol, total cholesterol, non-HDL-cholesterol, and apolipoprotein B. The study also provided evidence of favourable gut microbiota modulation, alongside an increase in vitamin D concentrations within the active group, which could be clinically relevant for the study population. These findings should be interpreted as preliminary, given the small sample size, short duration, and exploratory statistical framework. Nevertheless, they suggest that a combined probiotic–phytosterol approach may represent a promising adjunctive strategy to dietary management for lipid control in individuals with CeD, particularly for those seeking non-pharmacological options.

Together, these exploratory findings suggest coordinated metabolic and microbial changes that support further investigation of this probiotic–phytosterol formulation as a safe, natural adjunct to dietary management for lipid control in CeD. Confirmation in larger, adequately powered randomised controlled trials is now warranted.

## Figures and Tables

**Table 1:** Inclusion and exclusion criteria.

Inclusion criteria	Adults aged between 18 and 65 years, with a CeD diagnosis Strict adherence to a GFD as assessed by repeated negative anti-tissue transglutaminase IgA (anti-tTG IgA) and urine gluten immunogenic peptides (GIPs) in the clinical history and dietary interview Fasting blood glucose level between 5.6 and 6.9 mmol/L High baseline total cholesterol (>6 mmol/L) HbA1c below 5.7% For intervention purposes, eligible participants were also required to have a mobile phone and to speak Italian
Exclusion criteria	Failure to sign a consent form Age < 18 years or >65 years Increased levels of anti-TTG IgA and/or presence of GIPs in the urine in the clinical history. Gluten lapses in the dietary interview People with comorbid conditions that may limit participation in the study, such as a history of an acute cardiovascular event, uncontrolled hypertension, cancer, or major psychiatric or cognitive problems People who were already participating in a weight loss programme People receiving drug treatment for lipid metabolism (e.g., statins) People with a history of long-term use of medicines known to influence glucose metabolism (e.g., corticosteroids) People with elevated liver enzymes (alanine aminotransferase ≥ 300 IU/L, aspartate aminotransferase ≥ 300 IU/L) People who have taken antibiotics in the last 3 months or probiotics in the last month Pregnant women, women ready for pregnancy, and nursing mothers

**Table 2:** Baseline demographic and clinical characteristics of study participants in the active and placebo groups.

Variable	Placebo (*n* = 12)	Active (*n* = 12)	*p*
Age (years)	47.3 ± 7.9	47.75 ± 13.26	0.75
Gender (M/F)	2/10	6/6	
Height (cm)	164 ± 9.07	169 ± 10.73	0.52
Weight (kg)	66 ± 6.70	70 ± 15.53	0.32
BMI (kg/m^2^)	25 ± 6.0	26 ± 6.1	0.76

Data are expressed as Mean (± SD, standard deviation) for continuous variables and counts for categorical variables. *p* values were obtained using independent samples *t*-tests for continuous variables and Fisher’s exact test for categorical variables.

**Table 3:** Biochemical markers over time by treatment group.

Biomarker	Group	Baseline	Week 5	Week 9	Week 12	*p* (Time × Group)	Reference Value
**Total protein**	Placebo	6.76 ± 0.47	6.32 ± 0.35	6.60 ± 0.56	6.46 ± 0.40	0.834	6.0–8.3 g/dL
	Treatment	7.00 ± 0.41	6.77 ± 0.31	6.92 ± 0.81	6.87 ± 0.32		
**AST**	Placebo	24.50 ± 7.51	25.75 ± 7.18	20.75 ± 6.55	22.75 ± 3.30	0.401	10–40 U/L
	Treatment	24.00 ± 3.35	20.50 ± 3.73	19.33 ± 1.21	23.17 ± 4.92		
**ALT**	Placebo	17.80 ± 9.07	19.00 ± 10.12	14.60 ± 7.27	14.00 ± 5.24	0.333	7–56 U/L
	Treatment	14.83 ± 3.43	10.83 ± 2.79	10.50 ± 1.87	14.00 ± 8.03		
**Insulin**	Placebo	4.03 ± 0.74	6.63 ± 2.51	5.30 ± 0.90	5.60 ± 1.35	0.214	2–25 µIU/mL (fasting)
	Treatment	7.70 ± 2.80	5.20 ± 1.70	5.33 ± 2.45	6.58 ± 6.62		
**Albumin**	Placebo	4.18 ± 0.30	4.03 ± 0.25	4.10 ± 0.51	4.03 ± 0.32	0.778	3.5–5.0 g/dL
	Treatment	4.22 ± 0.23	3.98 ± 0.35	4.06 ± 0.29	4.12 ± 0.18		
**Bilirubin**	Placebo	0.45 ± 0.06	0.46 ± 0.11	0.45 ± 0.01	0.45 ± 0.09	0.160	0.1–1.2 mg/dL
	Treatment	0.46 ± 0.16	0.47 ± 0.14	0.49 ± 0.13	0.62 ± 0.22		

Values are presented as Mean ± Standard Deviation (SD). *p* values represent repeated-measures ANOVA time × group interaction. Reference values are based on standard adult clinical laboratory ranges and may vary by institution.

**Table 4:** Summary of repeated-measures ANOVA results for biochemical markers.

Biomarker	Time Effect F(df)	*p*	η^2^p	Group Effect F(df)	*p*	η^2^p	Time × Group F(df)	*p*	η^2^p
**Vitamin D**	F(2.81, 19.66) = 3.69	0.032	0.345	F(1, 7) = 0.04	0.851	0.005	F(2.81, 19.66) = 2.63	0.081	0.273
**Total Protein**	F(1.96, 17.62) = 1.69	0.213	0.158	F(1, 9) = 2.54	0.146	0.220	F(1.96, 17.62) = 0.18	0.834	0.019
**AST**	F(2.62, 20.94) = 2.09	0.138	0.207	F(1, 8) = 0.57	0.472	0.066	F(2.62, 20.94) = 1.01	0.401	0.112
**ALT**	F(2.59, 23.33) = 1.04	0.386	0.103	F(1, 9) = 1.81	0.211	0.167	F(2.59, 23.33) = 1.18	0.333	0.116
**Insulin**	F(1.48, 7.38) = 0.19	0.770	0.036	F(1, 5) = 0.16	0.704	0.031	F(1.48, 7.38) = 1.91	0.214	0.276
**Albumin**	F(1.74, 12.17) = 1.23	0.321	0.149	F(1, 7) = 0.01	0.936	0.001	F(1.74, 12.17) = 0.22	0.778	0.030
**Bilirubin**	F(2.14, 19.28) = 1.97	0.165	0.179	F(1, 9) = 0.60	0.460	0.062	F(2.14, 19.28) = 2.00	0.160	0.182

Values represent results of repeated-measures ANOVA with Greenhouse–Geisser correction applied where appropriate. Effect size is reported as partial eta squared (η^2^p).

**Table 5:** Average dietary intake of the participants.

Nutrient per Day	Placebo (n = 12)	Active (n = 12)	*p* Value
Protein (g)	65.67 (7.7)	89.19 (48.25)	0.2
Carbohydrate (g)	188 (47)	228.79 (64.77)	0.2
Fat (g)	90.68 (10.8)	92.1 (20.8)	0.8
Sugars only (g)	50.48 (15.9)	72.9 (36.58)	0.15
Fibre (g)	18.5 (2.86)	22 (10.8)	0.4
Saturated fatty acids (g)	25.9 (7.1)	25.45 (9.945)	0.9
Cholesterol (mg)	201(77.5)	333 (243)	0.19
Total daily energy intake (kcal)	1850 (245)	2117 (492)	0.2

Data are presented as Mean ± Standard Deviation. *p* values were obtained using an independent samples *t*-test comparing the placebo and active groups. Dietary fibre intake was below recommended levels in both groups, which is consistent with previous observations in individuals adhering to a gluten-free diet, where the exclusion of major cereal-based food groups may contribute to reduced fibre intake.

## Data Availability

The clinical data presented in this study are available on reasonable request from the corresponding author. The microbiota sequencing data are available at NCBI SRA under the BioProject ID PRJNAxxx.

## References

[1] Ludvigsson J.F., James S., Askling J., Stenestrand U., Ingelsson E. (2011). Nationwide cohort study of risk of ischemic heart disease in patients with celiac disease. Circulation.

[2] Al-Hussaini A., Alharthi H., Osman A., Eltayeb-Elsheikh N., Chentoufi A. (2018). Genetic susceptibility for celiac disease is highly prevalent in the Saudi population. Saudi J. Gastroenterol..

[3] Abadie V., Han A.S., Jabri B., Sollid L.M. (2024). New Insights on Genes, Gluten, and Immunopathogenesis of Celiac Disease. Gastroenterology.

[4] Saturni L., Ferretti G., Bacchetti T. (2010). The gluten-free diet: Safety and nutritional quality. Nutrients.

[5] Krupa-Kozak U., Drabińska N. (2016). Calcium in Gluten-Free Life: Health-Related and Nutritional Implications. Foods.

[6] Regula J., Cerba A., Suliburska J., Tinkov A.A. (2018). In Vitro Bioavailability of Calcium, Magnesium, Iron, Zinc, and Copper from Gluten-Free Breads Supplemented with Natural Additives. Biol. Trace Elem. Res..

[7] Fry L., Madden A.M., Fallaize R. (2018). An investigation into the nutritional composition and cost of gluten-free versus regular food products in the UK. J. Hum. Nutr. Diet..

[8] Jeanes Y., Spitale A., Nicolini G., Bergmann V., Fagbemi L., Rasheid R., Hovland C., Costabile A. (2022). Calcium and Iron Content of Cereal-Based Gluten-Free Products. Foods.

[9] Ciacci C., Cirillo M., Giorgetti G., Alfinito F., Franchi A., Mazzetti di Pietralata M., Mazzacca G. (1999). Low plasma cholesterol: A correlate of nondiagnosed celiac disease in adults with hypochromic anemia. Am. J. Gastroenterol..

[10] Tortora R., Capone P., De Stefano G., Imperatore N., Gerbino N., Donetto S., Monaco V., Caporaso N., Rispo A. (2015). Metabolic syndrome in patients with coeliac disease on a gluten-free diet. Aliment. Pharmacol. Ther..

[11] Zanini B., Caselani F., Magni A., Turini D., Ferraresi A., Lanzarotto F., Villanacci V., Ricci C., Lanzini A. (2013). Impact of gluten-free diet on cardiovascular risk factors. A retrospective analysis in a large cohort of coeliac patients. Dig. Liver Dis..

[12] Tetzlaff W., Tan M., Soliman M.L., Alhassan E., Kim H.S., Makharia G.K., Ramakrishna B.S., Makharia A. (2017). Markers of inflammation and cardiovascular disease in recently diagnosed celiac disease patients. World J. Cardiol..

[13] Agarwal A., Singh A., Mehtab W., Gupta V., Makharia G.K. (2021). Patients with celiac disease are at high risk of developing metabolic syndrome and fatty liver. Intest. Res..

[14] Ciccocioppo R., Kruzliak P., Cangemi G.C., Pohanka M., Betti E., Lauret E., Rodrigo L. (2015). The spectrum of differences between childhood and adulthood celiac disease. Nutrients.

[15] Dogra S.K., Doré J., Damak S. (2020). Gut microbiota resilience: Definition, link to health and strategies for intervention. Front. Microbiol..

[16] Leonard M.M., Serena G., Karathia H., Camhi S., Fritsch J., Sapone A., Senger S., Cucchiara S., Malamut G., Edwards J.A. (2021). Microbiome signatures of progression toward celiac disease onset in at-risk children in a longitudinal prospective cohort study. Proc. Natl. Acad. Sci. USA.

[17] Li X., Jann N.J., Yang Z.W., Al-Salami H., Sun J., Wang C.S., Gao P.H., Xu M.Q., Cui F.A., Chang M.L. (2020). Gut microbiota as an “invisible organ” that modulates the function of drugs. Biomed. Pharmacother..

[18] Catassi G., Lener E., Grattagliano M.M., Motuz S., Zavarella M.A., Bibbò S., Cammarota G., Gasbarrini A., Ianiro G., Catassi C. (2024). The role of microbiome in the development of gluten-related disorders. Best Pract. Res. Clin. Gastroenterol..

[19] Seiler C.L., Kiflen M., Stefanolo J.P., Bai J., Bercik P., Kelly C., Verdu E., Moayyedi P., Pinto-Sanchez M.I. (2020). Probiotics for celiac disease: A systematic review and meta-analysis of randomized controlled trials. Am. J. Gastroenterol..

[20] Rubio-Tapia A., Murray J.A. (2010). Classification and management of refractory coeliac disease. Gut.

[21] Fagbemi L.O., Soldaini C., Costabile A., Kolida S., Ciacci C., Jeanes Y. (2024). Probiotic interventions in coeliac disease: A systematic review with a focus on cardiovascular risk. Gastrointest. Disord..

[22] Costabile A., Buttarazzi I., Kolida S., Quercia S., Baldini J., Swann J.R., Brigidi P., Gibson G.R. (2017). An in vivo assessment of the cholesterol-lowering efficacy of Lactobacillus plantarum ECGC 13110402 in normal to mildly hypercholesterolaemic adults. PLoS ONE.

[23] Keleszade E., Kolida S., Costabile A. (2022). The cholesterol lowering efficacy of Lactobacillus plantarum ECGC 13110402 in hypercholesterolemic adults: A double-blind, randomized, placebo controlled, pilot human intervention study. J. Funct. Foods.

[24] Arnett D.K., Blumenthal R.S., Albert M.A., Buroker A.B., Goldberger Z.D., Hahn E.J., Himmelfarb C.D., Khera A., Lloyd-Jones D., McEvoy J.W. (2019). 2019 ACC/AHA Guideline on the Primary Prevention of Cardiovascular Disease: A Report of the American College of Cardiology/American Heart Association Task Force on Clinical Practice Guidelines. Circulation.

[25] Demonty I., Ras R.T., van der Knaap H.C.M., Duchateau G.S.M.J.E., Meijer L., Zock P.L., Geleijnse J.M., Trautwein E.A. (2009). Continuous Dose-Response Relationship of the LDL-Cholesterol–Lowering Effect of Phytosterol Intake. J. Nutr..

[26] Gylling H., Plat J., Turley S., Ginsberg H.N., Ellegård L., Jessup W., Jones P.J., Lütjohann D., Maerz W., Masana L. (2014). Plant sterols and plant stanols in the management of dyslipidaemia and prevention of cardiovascular disease. Atherosclerosis.

[27] Barkas F., Nomikos T., Liberopoulos E., Klouras E., Anastasiou G., Filippas-Ntekouan S., Elisaf M., Rizos E.C. (2023). Plant Sterols and Plant Stanols in Cholesterol Management and Cardiovascular Prevention. Nutrients.

[28] EFSA Panel on Dietetic Products, Nutrition and Allergies (NDA) (2012). Scientific Opinion on the substantiation of a health claim related to 3 g/day plant sterols/stanols and lowering blood LDL-cholesterol and reduced risk of (coronary) heart disease pursuant to Article 19 of Regulation (EC) No 1924/2006. EFSA J..

[29] Lohman T.G., Roche A.F., Martorell R. (1988). Anthropometric Standardization Reference Manual.

[30] World Health Organization (2010). WHO Guidelines on Drawing Blood: Best Practices in Phlebotomy.

[31] Costabile A., Corona G., Sarnsamak K.K., Atar-Zwillenberg D., Yit C., King A.J., Vauzour D., Barone M., Turroni S., Brigidi P. (2022). Wholegrain fermentation affects gut microbiota composition, phenolic acid metabolism and pancreatic beta cell function in a rodent model of type 2 diabetes. Front. Microbiol..

[32] Klindworth A., Pruesse E., Schweer T., Jörgensen J., Quast C., Horn M., Glöckner F.O. (2013). Evaluation of general 16S ribosomal RNA gene PCR primers for classical and next-generation sequencing-based diversity studies. Nucleic Acids Res..

[33] Fabbrini M. (2024). 16S-KrakenBracken-Pipeline (0.2.3) [Computer Program]. *GitHub*. https://github.com/FabbriniMarco/16S-KrakenBracken-Pipeline.

[34] Chen S., Zhou Y., Chen Y., Gu J. (2018). Fastp: An ultra-fast all-in-one FASTQ preprocessor. Bioinformatics.

[35] Quast C., Pruesse E., Yilmaz P., Gerken J., Schweer T., Yarza P., Peplies J., Glöckner F.O. (2013). The SILVA ribosomal RNA gene database project: Improved data processing and web-based tools. Nucleic Acids Res..

[36] Wood D.E., Lu J., Langmead B. (2019). Improved metagenomic analysis with Kraken 2. Genome Biol..

[37] Lu J., Salzberg S.L. (2020). Ultrafast and accurate 16S rRNA microbial community analysis using Kraken 2. Microbiome.

[38] Yang S. (2018). Summarizing OTU Table Regarding the Composition, Abundance and Beta Diversity of Abundant and Rare Biospheres (0.1.2) [Computer Program]. *GitHub*. https://github.com/cam315/otuSummary.

[39] McMurdie P.J., Holmes S. (2013). Phyloseq: An R Package for Reproducible Interactive Analysis and Graphics of Microbiome Census Data. PLoS ONE.

[40] Fabbrini M., Conti G. (2024). microbAIDeR—An Ensemble of Functions for Easier and Quicker Preliminary Microbiome Analyses (0.3.0) [Computer Program]. *GitHub*. https://github.com/FabbriniMarco/microbAIDeR/.

[41] Wickham H. (2016). ggplot2: Elegant Graphics for Data Analysis.

[42] Public Health England (2016). Government Dietary Recommendations Government Recommendations for Energy and Nutrients for Males and Females Aged 1–18 Years and 19+ Years.

[43] National Diet and Nutrition Survey (NDNS) (2019). Results from Years 9 to 11.

[44] Scientific Advisory Committee on Nutrition (SACN) (2015). Carbohydrates and Health Report.

[45] World Health Organization (2003). Diet, Nutrition and the Prevention of Chronic Diseases.

[46] Reynolds A., Mann J., Cummings J., Winter N., Mete E., Te Morenga L. (2019). Carbohydrate quality and human health: A series of systematic reviews and meta-analyses. Lancet.

[47] Anderson J.W., Baird P., Davis R.H., Ferreri S., Knudtson M., Koraym A., Waters V., Williams C.L. (2019). Health benefits of dietary fiber. Nutr. Rev..

[48] Zingone F., Ciacci C. (2018). The value and significance of 25(OH) and 1,25(OH) vitamin D serum levels in adult coeliac patients: A review of the literature. Dig. Liver Dis..

[49] Holick M.F. (2007). Vitamin D deficiency. N. Engl. J. Med..

[50] Cashman K.D., Dowling K.G., Škrabáková Z., Gonzalez-Gross M., Valtueña J., De Henauw S., Moreno L., Damsgaard C.T., Michaelsen K.F., Mølgaard C. (2016). Vitamin D deficiency in Europe: Pandemic?. Am. J. Clin. Nutr..

[51] Begley M., Hill C., Gahan C.G. (2006). Bile salt hydrolase activity in probiotics. Appl. Environ. Microbiol..

[52] Nguyen T.D.T., Kang J.H., Lee M.S. (2007). Characterization of *Lactobacillus plantarum* PH04, a potential probiotic bacterium with cholesterol-lowering effects. Int. J. Food Microbiol..

[53] Ciacci C., Cirillo M., Cavallaro R., Mazzacca G. (2002). Long-term follow-up of celiac adults on gluten-free diet: Prevalence and correlates of intestinal damage. Digestion.

[54] Vici G., Camilletti D., Polzonetti V. (2020). Possible role of vitamin D in celiac disease onset. Nutrients.

[55] Franks S.J., Dunster J.L., Carding S.R., Lord J.M., Hewison M., Calder P.C., King J.R. (2024). Modelling the influence of vitamin D and probiotic supplementation on the microbiome and immune response. Math. Med. Biol..

[56] Francavilla R., Piccolo M., Francavilla A., Polimeno L., Semeraro F., Cristofori F., Castellaneta S., Barone M., Indrio F., Gobbetti M. (2019). Clinical and Microbiological Effect of a Multispecies Probiotic Supplementation in Celiac Patients With Persistent IBS-type Symptoms: A Randomized, Double-Blind, Placebo-controlled, Multicenter Trial. J. Clin. Gastroenterol..

[57] Soheilian Khorzoghi M., Rostami-Nejad M., Yadegar A., Dabiri H., Hadadi A., Rodrigo L. (2023). Impact of probiotics on gut microbiota composition and clinical symptoms of coeliac disease patients following gluten-free diet. Contemp. Clin. Trials Commun..

[58] Belei O., Jugănaru I., Basaca D.G., Munteanu A.I., Mărginean O. (2023). The Role of Intestinal Microbiota in Celiac Disease and Further Therapeutic Perspectives. Life.

[59] Koh A., De Vadder F., Kovatcheva-Datchary P., Bäckhed F. (2016). From Dietary Fiber to Host Physiology: Short-Chain Fatty Acids as Key Bacterial Metabolites. Cell.

[60] Waters J.L., Ley R.E. (2019). The human gut bacteria *Christensenellaceae* are widespread, heritable, and associated with health. BMC Biol..

[61] Lim M.Y., You H.J., Yoon H.S., Kwon B., Lee J.Y., Lee S., Song Y.M., Lee K., Sung J., Ko G. (2017). The effect of heritability and host genetics on the gut microbiota and metabolic syndrome. Gut.

[62] Mancabelli L., Milani C., Lugli G.A., Turroni S., Ferrario C., van Sinderen D., Ventura M. (2017). Identification of universal gut microbial biomarkers of common human intestinal diseases by meta-analysis. FEMS Microbiol. Ecol..

[63] Chambers E.S., Preston T., Frost G., Morrison J.D. (2018). Role of Gut microbiota-generated short-chain fatty acids in metabolic and cardiovascular health. Curr. Nutr. Rep..

[64] Huang R., Zhou G., Cai J., Cao C., Zhu Z., Wu Q., Zhang F., Ding Y. (2025). Regulation of sterol metabolism by gut microbiota and its relevance to disease. Gut Microbes.

[65] Xie R., Guo Z., Gan H., He Q., Huang S., Yin M., Tang C., Wu X., Chen S., Huang X. (2025). Effects of Phytosterols on Growth Performance, Serum Indexes, and Fecal Microbiota in Finishing Pigs. Animals.

[66] Gao J., Lv J.X., Xie G.Q., Lv Y.C., Zheng W.T., Chang G.L., Sheng X.Y., Wang C. (2024). Dietary phytosterols improves the metabolic status of perinatal cows as evidenced by plasma metabolomics and faecal microbial metabolism. Anim. Biosci..

[67] Miszczuk E., Głąb S., Łaszkiewicz-Tomanek M., Ciesielska A., Szymańska E. (2024). Phytosterols and the Digestive System: A Review Study from Insights into Their Potential Health Benefits and Safety. Pharmaceuticals.

[68] Lee S.H., Kim S.Y., Kim H.W., Ryoo S., Oh H.J., Lee J.H., Kang K., Myung H.J. (2022). High Dose Intramuscular Vitamin D3 Supplementation Impacts the Gut Microbiota of Patients With Clostridioides Difficile Infection. Front. Cell. Infect. Microbiol..

[69] Bellerba F., Muirhead V., Cortese F., D’Auria G., Marzorati M., Licata M., Pala V., Gnagnarella P. (2021). The Association between Vitamin D and Gut Microbiota: A Systematic Review of Human Studies. Nutrients.

[70] Homma Y., Ikeda I., Ishikawa T., Tateno M., Sugano M., Nakamura H. (2003). Decrease in plasma low-density lipoprotein cholesterol, apolipoprotein B, cholesteryl ester transfer protein, and oxidized low-density lipoprotein by plant stanol ester-containing spread: A randomized, placebo-controlled trial. Nutrition.

[71] Madsen M.B., Jensen A.M., Schmidt E.B. (2007). The effect of a combination of plant sterol-enriched foods in mildly hypercholesterolemic subjects. Clin. Nutr..

[72] Richardson T.G., Sanderson E., Palmer T.M., Ala-Korpela M., Davey Smith G., Holmes M.V. (2021). Effects of apolipoprotein B on lifespan and risks of major diseases including type 2 diabetes: A mendelian randomisation analysis using outcomes in first-degree relatives. Lancet Healthy Longev..

[73] Valitutti F., Cavalli E., Leter B., Leonard M.M., Alessio F., Cucchiara S. (2025). Coeliac disease and microbiota: Is it time for personalised biotics intervention? A scoping review. BMJ Nutr. Prev. Health.

[74] Rondanelli M., Borromeo S., Cavioni A., Gasparri C., Gattone I., Genovese E., Lazzarotti A., Minonne L., Moroni A., Patelli Z. (2025). Therapeutic Strategies to Modulate Gut Microbial Health: Approaches for Chronic Metabolic Disorder Management. Metabolites.

[75] Eng S., Gabr A., Raghav S., Frishman W.H., Aronow W.S. (2025). The Gut-Heart Connection: Unraveling Cardiovascular Risks in Celiac Disease. Cardiol. Rev..

[76] Vaghela P., Dave B., Dabhade A., Deshmukh R., Prajapati B., Alsaidan O.A., Patel S., Mehta A., Singh A., Dudhat K. (2026). Redrawing the gut map: Evolving probiotic approaches to microbiota modulation in inflammatory bowel disease. Antonie Van. Leeuwenhoek.

